# Overview of the Cancer Genetics and Pathway Curation tasks of BioNLP Shared Task 2013

**DOI:** 10.1186/1471-2105-16-S10-S2

**Published:** 2015-07-13

**Authors:** Sampo Pyysalo, Tomoko Ohta, Rafal Rak, Andrew Rowley, Hong-Woo Chun, Sung-Jae Jung, Sung-Pil Choi, Jun'ichi Tsujii, Sophia Ananiadou

**Affiliations:** 1 Department of Information technology, University of Turku, Turku, Finland; 2 textimi, Tokyo, Japan; 3 National Centre for Text Mining and School of Computer Science, University of Manchester, Manchester, UK; 4 Software Research Center, Korea Institute of Science and Technology Information (KISTI), Daejeon, South Korea; 5 Department of Applied Information Science, University of Science and Technology (UST), Daejeon, South Korea; 6 Department of Library and Information Science, Kyonggi University, Suwon, South Korea; 7 Microsoft Research Asia, Beijing, China

## Abstract

**Background:**

Since their introduction in 2009, the BioNLP Shared Task events have been instrumental in advancing the development of methods and resources for the automatic extraction of information from the biomedical literature. In this paper, we present the Cancer Genetics (CG) and Pathway Curation (PC) tasks, two event extraction tasks introduced in the BioNLP Shared Task 2013. The CG task focuses on cancer, emphasizing the extraction of physiological and pathological processes at various levels of biological organization, and the PC task targets reactions relevant to the development of biomolecular pathway models, defining its extraction targets on the basis of established pathway representations and ontologies.

**Results:**

Six groups participated in the CG task and two groups in the PC task, together applying a wide range of extraction approaches including both established state-of-the-art systems and newly introduced extraction methods. The best-performing systems achieved F-scores of 55% on the CG task and 53% on the PC task, demonstrating a level of performance comparable to the best results achieved in similar previously proposed tasks.

**Conclusions:**

The results indicate that existing event extraction technology can generalize to meet the novel challenges represented by the CG and PC task settings, suggesting that extraction methods are capable of supporting the construction of knowledge bases on the molecular mechanisms of cancer and the curation of biomolecular pathway models. The CG and PC tasks continue as open challenges for all interested parties, with data, tools and resources available from the shared task homepage.

## Background

The BioNLP Shared Task (BioNLP ST), organized for the third time in 2013, presents open challenges in biomedical natural language processing to all interested parties. The shared task organizers provide task definitions, manually annotated data for method development and evaluation, and tools for the assessment and comparison of information extraction approaches proposed by the community [1-3]. We describe two of the event extraction tasks of the BioNLP ST'13 challenge, the Cancer Genetics (CG) and Pathway Curation (PC) tasks, both of which were newly introduced for the 2013 challenge. This manuscripts extends on the previous papers presenting the tasks at the BioNLP ST workshop [4,5].

Both the CG and PC tasks aim to support *knowledge base construction*, the over-arching theme of the 2013 BioNLP ST. That is, both tasks are motivated by the need to develop better methods for automatically analyzing the literature at large scale in order to support the creation, maintenance and further development of structured representations of domain knowledge. However, the specific representations and subdomains of biomedical knowledge targeted by the tasks differ substantially, leading to two different information extraction task settings.

The CG task targets the automatic analysis of the literature on cancer, a complex group of genetic diseases that is one of the most common causes of death worldwide. Despite decades of focused research and an ever-increasing body of knowledge on its causes and mechanisms, cancer remains imperfectly understood [6,7]. Part of the challenge of building comprehensive knowledge bases on cancer is the sheer volume of information on the topic: for example, a search for "*cancer*" in the PubMed literature database [8] finds mentions in over three million publications, or in approximately one in every eight papers published in the domain.

By contrast to the disease-oriented focus of the CG task, the goals of the PC task are defined by a specific category of knowledge representation, namely formal models of biomolecular pathways such as the Systems Biology Markup Language (SBML) [9,10] and the Biological Pathway Exchange (BioPAX) language [11,12]. Structured models such as these are increasingly applied to capture the best current understanding of complex biomolecular systems and represent it in a way that permits automatic processing [13], and a large number of such models can be found in repositories such as BioModels [14] and PANTHER DB [15]. However, the construction of these models is a demanding task, with large models potentially combining information from thousands of individual publications from among the millions of published papers involving biomolecules and their reactions.

Although cancer knowledge base construction and pathway model curation both face challenges stemming from the enormous size and rapid growth of the biomedical literature, existing tools for the automatic analysis of that literature have not fully addressed the needs of these important tasks. In particular, in biomedical information extraction there has long been considerable focus on the recognition of biomolecular entities and binary relations such as protein-protein interaction involving these entities [16-20], largely to the exclusion of upper levels of biological organization and richer representations of entity relations such as pathway models.

While previous BioNLP ST events have been instrumental in promoting the extraction of more expressive representations in the form of event structures [21,22], they have still been mostly limited to molecular and subcellular level entities and processes (Table 1). As an understanding of cancer requires the ability to associate molecular level causes with cellular, tissue- and organ-level effects and organism-level outcomes, methods following the approaches of these previous shared tasks are only capable of addressing a modest part of the challenge of supporting cancer knowledge base construction. Similarly, although the event structures applied in the BioNLP ST tasks have similar expressivity to pathway models, former tasks in the series have not directly addressed either the specific representation nor the semantics of major pathway formalisms, leaving open significant challenges in mapping between event structures, pathway reactions, and the semantic types of the two [23,24].

**Table 1:** Properties of selected shared tasks in biomedical information extraction prior to the BioNLP ST 13.

Task	Levels of biological organization	Representation
LLL'05	Molecular	Binary relations
BioCreative II PPI	Molecular	Binary relations
BioCreative II.5 IPT	Molecular	Binary relations
BioNLP ST'09 GE	Molecular, subcellular	Event structures
BioCreative III PPI	Molecular	Binary relations
BioNLP ST'11 GE	Molecular, subcellular	Event structures
BioNLP ST'11 EPI	Molecular	Event structures
BioNLP ST'11 ID	Molecular, organism	Event structures
BioNLP ST'11 BB	Cellular, anatomical, environment	Binary relations
BioNLP ST'11 BI	Molecular	Event structures

Entity mention-focused tasks (detection and normalization) and the supporting tasks of the BioNLP ST are not included.

We believe that the event extraction approach can substantially benefit both cancer knowledge base construction and the curation of biomolecular pathway models, and the BioNLP ST'13 Cancer Genetics and Pathway Curation tasks aim to close the gaps hindering the realization of these benefits. Specifically, the CG task introduces novel extraction targets covering all levels of biological organization ranging from the molecular to the whole organism, further extending the scope of previous tasks in the series through the inclusion of pathological processes and events representing experimenter action. The PC task bases both its representation and its semantics directly on major pathway model standards such as SBML, BioPAX and the Systems Biology Ontology [25,26] to assure the compatibility of extracted information with these efforts. Extensive newly annotated corpora are introduced to support both of these novel extraction tasks.

In the following, we first present the applied event representation and its application in the CG and PC task settings in detail. We next introduce the corpora for the two tasks and the criteria for evaluating the predictions submitted by task participants against their annotation. We then present the task participants and systems, the primary evaluation results and additional analyses of these results, and finally discuss the overall findings of these tasks and present conclusions.

## Methods

### Representation

The BioNLP ST main tasks are information extraction tasks targeting *event structures *(or *events *for short) [27] following the general approach first introduced in the BioNLP ST'09 [28]. Events capture information on reactions and processes involving physical *entities *of interest. In addition to entities and events, the representation applied in the tasks involves *relations *between entities and *event modifications *that identify additional aspects of extracted events such as speculation and negation. We next present these annotation primitives, and then proceed to present the specific types and their definitions applied in the CG and PC tasks.

#### Entities

**Figure 1:**
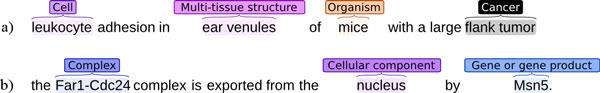
**Illustration of entity annotations**. a) Cancer Genetics task b) Pathway Curation task. (Illustrations created with BRAT[59])

Each mention of a relevant physical entity is annotated as a contiguous span of text that is assigned a type such as CELL or SIMPLE CHEMICAL from a closed set of entity types defined for the task. Figure 1 shows examples of entity annotation.

The recognition of mentions of physical entities in free text is a very well-studied task both in general-domain and biomedical natural language processing [29,30], and the recognition of many key entity types relevant to the CG and PC tasks has been considered in particular for molecular level entities in a number of tasks in the BioCreative series of community evaluations [16,17,31]. Thus, to focus the efforts of participants on the novel aspects of the CG and PC tasks, manually created ("gold standard") physical entity mention annotations are provided to participants also for test data, following a convention first established in the BioNLP ST'09.

#### Relations

**Figure 2:**
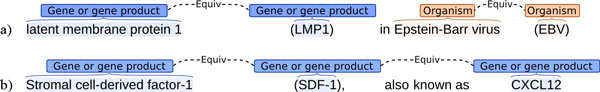
**Illustration of relation annotations**. a) Cancer Genetics task b) Pathway Curation task.

The CG and PC tasks both define a single relation type, *Equiv. Equiv *is a symmetric, transitive binary relation that identifies entity mentions as being equivalent in the sense of referring to the same real-world entity. *Equiv *relations are used to mark local aliases such as abbreviations. Figure 2 shows examples of the relation annotation.

The relation annotations are not an extraction target in the task, and gold standard *Equiv *annotation is applied also for the test data. The *Equiv *annotations are used in evaluation when determining if two events match: for event matching, any entities connected by an *Equiv *relation (directly or transitively) are interchangeable.

#### Events

Events, the primary extraction target in BioNLP ST main tasks, are structured annotations, each of which has a type, zero or more participants, and an associated statement in text that expresses the event (the *event trigger*). As for entity annotations, event types are drawn from a closed, task-specific set, and the event trigger is a contiguous span of characters. Event participants can be either entity or event annotations, the latter allowing complex event structures where one event is identified as e.g. causing or preventing another. For each event participant, the *role *that the participant plays in the event is further identified. The number of role types is small and mostly generic, but some moderately task-specific roles are also defined. The following roles are used in the CG and PC tasks:

***Theme ***Entity or event that undergoes the primary effects of the event. For example, the GENE OR GENE PRODUCT "*p53 *" in "*p53 is transcribed *" and the DEVELOPMENT event in "*regulation of . . . development*".

***Cause ***Entity or event that is causally active in the event. Example: the "*insulin*" entity in "*insulin regulates VEGF expression*" and the NEGATIVE REGULATION event triggered by "*inhibition*" in "*inhibition . . . prevented necrosis*".

***Participant ***Entity or event that participates in the event in a way that is not specified in detail in the context. Example: GENE OR GENE PRODUCT "*TAK1 *" in "*TAK1 is involved in Wnt signaling*".

***Product ***Entity that is produced in the event. Example: the COMPLEX "*NF-kappa B*" in "*formation of NF-kappa B*". Not applied in the CG task.

***Instrument ***Entity used to carry out the event. Example: the GENE OR GENE PRODUCT "*angiostatin*" in "*mice were treated with angiostatin*". Not applied in the PC task.

***Site ***Part of the *Theme *entity that is specifically affected by the event. Example: "*serine-15*" in "*phosphorylation of p53 on serine-15*"

***AtLoc ***Location where the event takes place. Example: the ORGAN "*skin*" in "*skin tumorigenesis*" and the CELLULAR COMPONENT "*nucleus*" in "*Rlm1 resides in the nucleus*".

***FromLoc ***Source of movement in events involving change of location. Example: the CELLULAR COMPONENT "*mitochondria*" in "*release of cytochrome c from the mitochondria*" and "*cytoplasm*" in "*MAPK translocates from the cytoplasm*"

***ToLoc ***Direction or end point of movement in events involving change of location. Example: "*lymph node*" in "*lymph node metastasis*" and "*plasma membrane*" in "*RSK1 translocates to the plasma membrane*".

**Figure 3:**
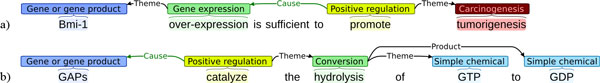
**Illustration of event annotations**. a) Cancer Genetics task b) Pathway Curation task.

Examples of event annotation are shown in Figure 3.

#### Event modifications

Both the CG and PC tasks involve the modification types NEGATION and SPECULATION. These modifications are simple binary flags that apply to an event, marking it as being explicitly denied (NEGATION , e.g. "*p53 is not affected *") or expressed in a speculative context (SPECULATION, e.g. "*p53 may be affected*"). Event modifications are not associated with a text "trigger", unlike in some related task settings [32,33]. The representation, annotation scope, and semantics of these modifications are defined as in the BioNLP ST'09, similarly to many other subsequent tasks. Event modifications are one of the extraction targets in the two tasks, and systems aiming to address all aspects of the tasks should identify also event NEGATION and SPECULATION.

#### Data format

**Figure 4:**
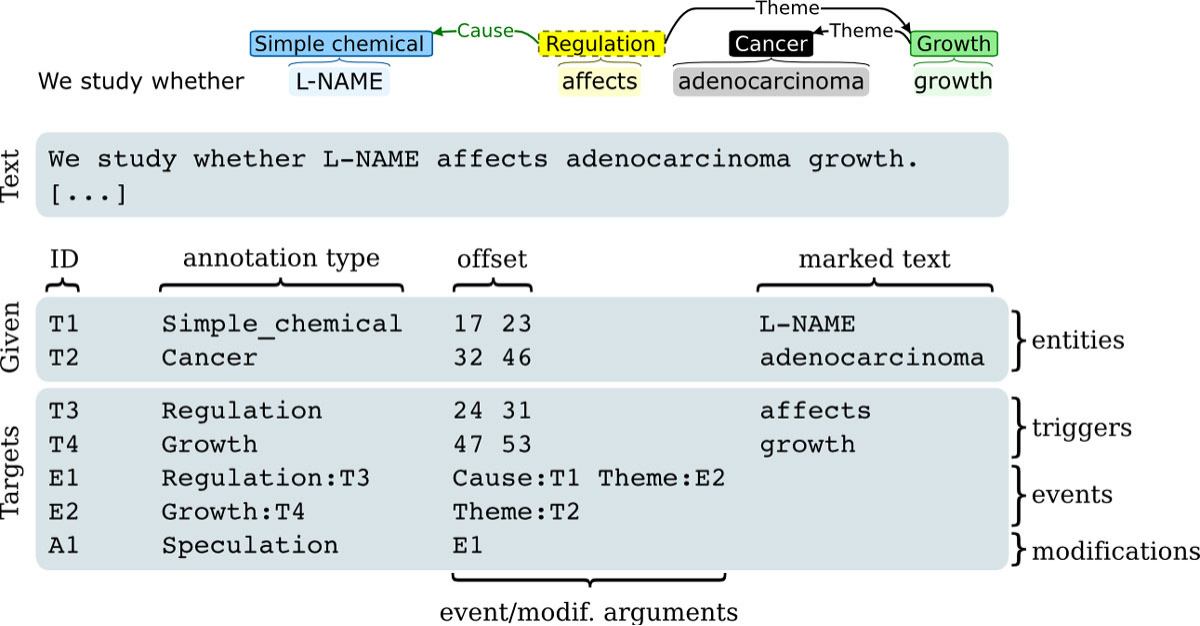
**Illustration of the data format**. Adapted from [36].

The information introduced above is represented in the tasks using the simple data format first introduced for the BioNLP ST'09. This is a standoff format where the annotated texts are stored separately from their annotations, with annotations referencing relevant spans of text via character offsets. The annotations are further split so that the physical entity annotations that are provided to the task participants also for the test data are stored in one file, and the annotations corresponding to extraction targets in another. Each document thus involves three files: the text, the given annotations, and the extraction targets. The annotations are stored in a line-oriented format where each line contains a single annotation, consisting of a unique identifier, the annotation type, and fields specific to the annotation primitive, such as the trigger and event arguments for event annotations, or the (*start, end*) character offsets and marked text for physical entity annotations and event triggers. Figure 4 shows a simple example illustrating the data format. For a detailed specification, we refer to the documentation on the shared task homepage [34].

### Cancer Genetics task setting

Table 2 summarizes the entity types and reference resources applied in the CG task annotation. Of the molecular level entity types, the most prominent are SIMPLE CHEMICAL, used to mark mentions of the names of non-repetitive chemical entities, and GENE OR GENE PRODUCT, used for the names of genes, proteins, RNA, and their families. These two entity types are closely similar in definition to the CHEMICAL and PROTEIN types included in previous ST tasks [35,36]. However, the CG molecular types are more fine-grained than the corresponding types in these previous tasks, and the task applies detailed types such as PROTEIN DOMAIN OR REGION for cases where many previous tasks used the generic type ENTITY[28].

**Table 2:** Cancer Genetics task entity types, reference resources, and definitions.

Type	Reference	Ontology ID
ORGANISM	NCBI taxonomy	CARO:0000012
*Anatomical entity*		
ORGANISM SUBDIVISION	Species-specific	CARO:0000032
ANATOMICAL SYSTEM	anatomy	CARO:0000011
ORGAN	resources (e.g.	CARO:0000024
MULTI-TISSUE STRUCTURE	FMA), derived	CARO:0000055
TISSUE	resources (e.g.	CARO:0000043
DEVELOPING ANATOMICAL STRUCTURE	UBERON)	UBERON:0005423
CELL	CL	CARO:0000013
CELLULAR COMPONENT	GO-CC	GO:0005575
ORGANISM SUBSTANCE	FMA etc.	CARO:0000004
IMMATERIAL ANATOMICAL ENTITY	FMA etc.	CARO:0000007
PATHOLOGICAL FORMATION	-	-
CANCER	-	-
*Molecular entity*		
GENE OR GENE PRODUCT	gene, protein,	SBO:0000246
PROTEIN DOMAIN OR REGION	and related	SBO:0000493
DNA DOMAIN OR REGION	entity DBs	SBO:0000493
SIMPLE CHEMICAL	ChEBI	SBO:0000247
AMINO ACID	ChEBI	CHEBI:33709

The indentation of the types corresponds to *is-a *relations. The labels in *italics *are not annotated types but groupings defined only for organization.

Anatomy-level entities, a notable novel aspect of the CG task, are subdivided primarily by granularity, and their type labels and definitions mainly follow the Common Anatomy Reference Ontology (CARO) [37], which is a small species-independent ontology based on the Foundational Model of Anatomy (FMA) [38]. For anatomical entities, not only names but also nominal and adjectival references (e.g. "*mitochondrial*") are annotated. We refer to Ohta et al. [39] for more detailed discussion of the anatomical entity type definitions. Finally, the names of organisms and nominal mentions of comparable specificity (e.g. "*patients*") are annotated as ORGANISM, as in a number of previous tasks.

To assure consistency, resources identifying entities of each relevant type were used for reference during the entity annotation. Mentions of biomolecules are annotated with reference to databases of genes, proteins, their families, and related entities such as Entrez Gene [40], UniProt [41], PFam [42], and CATH [43], and simple chemical entity mentions with reference to ChEBI [44]. Anatomical entity mentions are annotated primarily with reference to species-specific anatomy ontologies such as FMA and derived resources such as UBERON [45]. For cells and cellular components, available cross-species resources such as the Cell Ontology (CL) [46] and the Gene Ontology Cellular Component subontology (CO-CC) are used. ORGANISM annotations are created with reference to the NCBI taxonomy [47].

The CG event types and their arguments are summarized in Table 3. As in most previously introduced BioNLP ST task settings, the CG task bases its event types primarily on the Gene Ontology (GO) [48]. Specifically, the definitions of event types of the molecular, general and regulation categories follow the corresponding GO-based types in the 2011 GENIA (GE) [35] and Epigenetics and Post-translational Modifications (EPI) [36] tasks. By contrast, event types of the anatomical and pathological categories have not been considered in previous ST settings. As for the other categories, these events are defined with GO as the primary reference resource whenever possible.

**Table 3:** Cancer Genetics task event types and their arguments.

Type	Core arguments	Additional arguments
*Anatomical*		
DEVELOPMENT	*Theme *(Anatomy)	
BLOOD VESSEL DEVELOPMENT	*Theme*?(Anatomy)	*AtLoc?*
GROWTH	*Theme *(Anatomy)	
DEATH	*Theme *(Anatomy)	
CELL DEATH	*Theme*?(Cell)	
BREAKDOWN	*Theme *(Anatomy)	
CELL PROLIFERATION	*Theme *(Cell)	
CELL DIVISION	*Theme *(Cell)	
CELL DIFFERENTIATION	*Theme *(Cell)	*AtLoc?*
REMODELING	*Theme *(Tissue)	
REPRODUCTION	*Theme *(Organism)	
*Pathological*		
MUTATION	*Theme *(GGP)	*AtLoc?, Site?*
CARCINOGENESIS	*Theme*?(Anatomy)	*AtLoc?*
CELL TRANSFORMATION	*Theme *(Cell)	*AtLoc?*
METASTASIS	*Theme*?(Anatomy)	*ToLoc*
INFECTION	*Theme*?(Anatomy), *Participant*?(Organism)	
*Molecular*		
METABOLISM	*Theme *(Molecule)	
SYNTHESIS	*Theme *(Simple chemical)	
CATABOLISM	*Theme *(Molecule)	
AMINO ACID CATABOLISM	*Theme*?(Molecule)	
GLYCOLYSIS	*Theme*?(Molecule)	
GENE EXPRESSION	*Theme*+(GGP)	
TRANSCRIPTION	*Theme *(GGP)	
TRANSLATION	*Theme *(GGP)	
PROTEIN PROCESSING	*Theme *(GGP)	
PHOSPHORYLATION	*Theme *(Molecule)	*Site?*
(other chemical modifications defined similarly to PHOSPHORYLATION)		
PATHWAY	*Participant *(Molecule)	
*General*		
BINDING	*Theme*+(Molecule)	*Site?*
DISSOCIATION	*Theme *(Molecule)	*Site?*
LOCALIZATION	*Theme*+(Molecule)	*At/From/ToLoc?*
Regulation	*Theme *(Any), *Cause*?(Any)	
POSITIVE REGULATION	*Theme *(Any), *Cause*?(Any)	
NEGATIVE REGULATION	*Theme *(Any), *Cause*?(Any)	
PLANNED PROCESS	Theme*(Any), Instrument*(Entity)	

The indentation corresponds to ontological structure (*is-a*/*part-of *relations). The suffixes ?, *, and *+ *denote zero or one, zero or more, and one or more arguments of the shown type (respectively). GGP stands for Gene or gene product. For brevity, additional argument types are not shown in table: the *AtLoc, FromLoc *and *ToLoc *arguments take an anatomical entity type, and *Site *arguments take a Protein domain or region or DNA domain or region entity type.

However, there are some categories of processes that are critical for comprehensive analysis of the cancer literature but fall outside of the scope of GO. Most importantly, GO systematically excludes from its scope pathological processes such as carcinogenesis. We address this limitation in two ways. First, we reinterpret GO types such as GROWTH to extend their scope so as to include also pathological entities, so that e.g. "*cancer development*" is annotated with the GROWTH type. Second, for intrinsically pathological processes such as METASTASIS that lack a close physiological analogue, we introduce a small number of representative high-level event types such as Metastasis. Finally, we introduce the single upper-level event type PLANNED PROCESS to mark processes explicitly involving experimenter action [49]. We refer to [50] for further information on the design of the CG task events.

### Pathway Curation task setting

Four entity types are defined in the PC task: the molecular level entity categories SIMPLE CHEMICAL, GENE OR GENE PRODUCT, and COMPLEX, and the CELLULAR COMPONENT type (Table 4). Of these four, all but COMPLEX are defined also in the CG task (see previous section), and the two tasks share the definitions of these types. The remaining type, COMPLEX, is used to mark the names of molecular entities of non-covalently linked components, which are particularly relevant to the PC task extraction targets.

**Table 4:** Pathway Curation task entity types, reference resources, and definitions.

Entity type	Reference	Ontology ID
SIMPLE CHEMICAL	ChEBI	SBO:0000247
GENE OR GENE PRODUCT	gene/protein DBs	SBO:0000246
COMPLEX	complex DBs	SBO:0000253
CELLULAR COMPONENT	GO-CC	SBO:0000290

Table 5 presents the event types annotated in the PC task and their arguments. By contrast to the CG task and the majority of previously introduced BioNLP ST tasks, the PC task applies the Systems Biology Ontology (SBO) as its primary reference ontology for events, only basing a small number of upper-level event types on GO. While the use of SBO for the PC task entity and event definitions assures compatibility with pathway representations on the type level, supporting pathway curation tasks using event extraction further requires the ability to map event *structures *to complex pathway reactions (and vice versa).

**Table 5:** Pathway Curation task event types and arguments.

Event type	Core arguments	Additional arguments	Ontology ID
CONVERSION	*Theme*(Molecule), *Product*(Molecule)		SBO:0000182
PHOSPHORYLATION	*Theme*(Molecule), *Cause*(Molecule)	*Site*(Simple chemical)	SBO:0000216
DEPHOSPHORYLATION	*Theme*(Molecule), *Cause*(Molecule)	*Site*(Simple chemical)	SBO:0000330
(Other modifications, such as ACETYLATION, defined similarly.)			
LOCALIZATION	*Theme*(Molecule)	*At/From/ToLoc*(Cell. comp.)	GO:0051179
TRANSPORT	*Theme*(Molecule)	*From/ToLoc*(Cell. comp.)	SBO:0000185
GENE EXPRESSION	*Theme*(Gene or gene product)		GO:0010467
TRANSCRIPTION	*Theme*(Gene or gene product)		SBO:0000183
TRANSLATION	*Theme*(Gene or gene product)		SBO:0000184
DEGRADATION	*Theme*(Molecule)		SBO:0000179
BINDING	*Theme*(Molecule), *Product*(Complex)		SBO:0000177
DISSOCIATION	*Theme*(Complex), *Product*(Molecule)		SBO:0000180
REGULATION	*Theme*(Any), *Cause*(Any)		GO:0065007
POSITIVE REGULATION	*Theme*(Any), *Cause*(Any)		GO:0048518,
			GO:0044093
ACTIVATION	*Theme*(Molecule), *Cause*(Any)		SBO:0000412
NEGATIVE REGULATION	*Theme*(Any), *Cause*(Any)		GO:0048519,
			GO:0044092
INACTIVATION	*Theme*(Molecule), *Cause*(Any)		SBO:0000412
PATHWAY	*Participant*(Molecule)		SBO:0000375

"Molecule" represents any of Simple chemical, Gene or gene product, or Complex. "Any" refers to an annotation of any type. The indentation of the types corresponds to ontological relations (*is-a *and *part-of *) between the event types

Popular pathway model standards such as SBML and BioPAX differentiate between three basic categories of reaction participants: reactants, products, and modifiers. The former two correspond roughly to reaction inputs and outputs, while the last category identifies entities that have a regulatory effect, such as inhibiting or catalyzing the reaction. While this categorization of pathway reaction participants has some close analogies to the event structures applied in previous shared tasks, some aspects of the event representation require adjustments to assure a systematic and consistent mapping. In the PC task, we interpret event role types as follows: first, *Theme *is used to annotate participants corresponding to reactants, and *Cause *is applied together with regulation event types to capture modifiers and their effects (inhibition, catalysis, etc.). Finally, we introduce the new role *Product *specifically for the purpose of representing the product role in pathway models.

**Figure 5:**
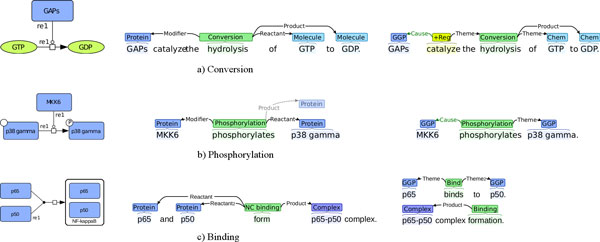
**Pathway model reactions and event representations**. Illustration of reactions in a pathway model (left), idealized explicit statements annotated with a directly mapped representation (center), and realistic expressions in text with actual event annotation. Figure from [5].

The use of SBO types and event roles defined with respect to the participant categories in pathways makes events and pathway reactions fully isomorphic in theory. However, natural language text does not necessarily explicitly state all participants, so in practice event representations that adhere to the principle that all annotations are bound to specific expressions in text may not always map to complete reaction representations. For example, in a pathway model, a phosphorylation reaction that takes *p38γ *as a reactant would have *phospho-p38γ *as a product. However, as the combination of reactant, reaction type and product is redundant, authors rarely express all three, instead using more concise forms such as "*p38γ *is phosphorylated". Figure 5 illustrates the mapping and the difference between idealized, fully explicit statements and less complete forms that frequently appear in actual publications. We refer to [24] for further discussion of the relation between the PC task entity and event types, the event representation, and representations applied in pathway models.

### Corpora

Both the Cancer Genetics and Pathway Curation tasks use corpus resources based in part on relevant previously released corpora. Nevertheless, most of the annotations in the shared task versions of the training, development and test corpora of both tasks were newly introduced specifically for these tasks. The following sections present these resources and their preparation.

#### Annotation process

The annotation of the CG and PC task corpora followed the same overall process: document selection, automatic pre-annotation of entity mentions, manual finalization of entity annotations, and manual event annotation.

While some of the aspects of the entity annotation are novel, many of the annotated entity types are in scope of established domain tools and resources. To reduce the overall annotation effort, we thus created preliminary annotation using a selection of automatic named entity and entity mention taggers. For SIMPLE CHEMICAL tagging, we used the OSCAR4 system, which was trained on the chemical entity mention recognition corpus of Corbett and Copestake [51]. For GENE OR GENE PRODUCT mention detection, we used BANNER[52] for the CG task and NERsuite [53] for the PC task. Both of these systems were trained on the Gene Mention task corpus introduced in the BioCreative 2 evaluation [54]. NERsuite was also applied for anatomical entity mention detection (CULLULAR COMPONENT only for the PC task). For these tagging tasks, the general machine learning-based system was trained on the Anatomical Entity Mention (AnEM) corpus [39] following the approach presented by Pyysalo and Ananiadou [55]. As no broad-coverage corpus annotated specifically for mentions of macromolecular complexes was available, we applied heuristics based on the GENE OR GENE PRODUCT annotation and dictionary-based tagging to create the initial annotations for the PC task COMPLEX type. Finally, LINNAEUS [56] was applied for the CG task ORGANISM mentions. The overall processing used the pipeline first introduced for similar analysis for the BioNLP ST'11 [57]. These tools were additionally integrated into the Argo workflow system [58] to support the PC task curation process. Following initial automatic entity mention annotation, we performed manual revision of the outputs to correct tagger errors prior to advancing to the event annotation stage.

We acknowledge that automatic annotation is not only far from perfect, but also carries a risk of introducing systematic errors, some of which may persist through subsequent manual revision. As entity annotations were not a target of extraction in either of the tasks, the possibility of some remaining bias from such errors was considered acceptable. By contrast, we wished to assure that the quality of the event annotations was as high as possible and to avoid any possibility of introducing systematic errors that might call into question whether the evaluation provides a fair representation of the comparative performance of different extraction approaches. For this reason, the event annotation of both tasks was created manually from scratch, forgoing any initial automatic annotation.

All manual annotation, including the revision of the initial automatic entity mention annotations as well as the primary event annotation, was performed using the open source BRAT annotation tool [59].

The task-specific annotation process details are presented below.

#### Cancer Genetics corpus annotation

The CG corpus consists of 600 PubMed abstracts selected on the basis of their relevance to established hallmarks of cancer, such as the evasion of apoptosis, tissue invasion, and sustained angiogenesis [6]. Of these 600 documents, 250 are part of the previously released multi-level event extraction (MLEE) corpus [50], which concentrates specifically on the angiogenesis subdomain. The other 350 documents were selected by querying PubMed for MeSH terms that relate to other hallmarks of cancer, such as apoptosis and metastasis (Table 6). After identifying queries providing sufficient specificity, we selected a random sample of the resulting documents and performed a round of manual filtering to assure that each abstract is relevant to the selected characteristic of cancer and its molecular basis.

**Table 6:** Queries for Cancer Genetics task document selection.

Domain	Documents	Query terms
Carcinogenesis	150	cell transformation, neoplastic AND (proteins OR genes)
Metastasis	100	neoplasm metastasis AND (proteins OR genes)
Apoptosis	50	apoptosis AND (proteins OR genes)
Glucose metabolism	50	(glucose/metabolism OR glycolysis) AND neoplasms

Only matches against MeSH terms were queried for, excluding cases where the query terms appeared only in text (e.g. "neoplasms"[MeSH Terms]).

The CG corpus was annotated by Tomoko Ohta, a PhD biologist with a cancer molecular biology background and extensive experience in event annotation. The creation of the anatomical entity annotation is described in [55], and the event annotation extended the guidelines and manual annotation process introduced in [50].

After the completion of the initial manual annotation, we performed some iterations of automatically supported revisions to further improve annotation quality and consistency. For example, we used the consistency check options of the search.py script included with BRAT to find text strings that were annotated as entity mentions in some cases but not in others and annotations that shared the same surface form but were assigned different semantic types. Automatically detected potential inconsistencies were subjected to further manual examination and correction when necessary. As only a single annotator was trained to perform the CG corpus annotation, we did not directly measure inter-annotator agreement during the annotation of this corpus. However, our previous IAA evaluation using the same annotation protocol [50] indicated that following an initial training round, the anatomical entity annotation could be replicated by a second annotator with 94.1% accuracy and the event annotation with 72.5% F-score using the primary task evaluation criteria.

#### Pathway Curation corpus annotation

As for the CG task, the texts of the PC task corpus are also drawn from PubMed abstracts. For the CG corpus, we aimed to assure that each included abstract provides evidence for the annotation of some reaction found in at least one selected pathway model (Table 7). To select such documents, we applied two complementary strategies, one predicated on the presence of explicit references to evidence documents in the pathway annotations, and the other using a system developed specifically to support the creation of such annotations.

**Table 7:** Pathway models used to select documents for the Pathway Curation task.

Pathway	Repository	ID	Publication
mTOR	BioModels	MODEL1012220002	[88]
mTORC1 upstream regulators	BioModels	MODEL1012220003	[88]
TLR	BioModels	MODEL2463683119	[89]
Yeast Cell Cycle	BioModels	MODEL1011020000	[90]
Rb	BioModels	MODEL4132046015	[91]
EGFR	BioModels	MODEL2463576061	[92]
Human Metabolic Network	BioModels	MODEL6399676120	[93]
NF-kappaB pathway	-	-	[23]
p38 MAPK	PANTHER DB	P05918	-
p53	PANTHER DB	P00059	-
p53 feedback loop pathway	PANTHER DB	P04392	-
Wnt signaling pathway	PANTHER DB	P00057	-

For the first approach, we selected the BioModels pathways that had the most manually annotated references identifying a document in PubMed. We then retrieved the abstracts of a random sample of these documents and filtered them manually to assure their relevance to the task. The second strategy was required as the majority of pathway models do not include any references to evidence documents. To select documents providing evidence for reactions in other pathways, we entered selected PANTHER DB models into PathText [60], a system for identifying documents relevant to specific reactions. We then selected random reactions from these models and manually filtered the top results retrieved by PathText for relevance. This approach is described in detail by Miwa et al. [61].

The PC task annotation effort was carried out in collaboration between the UK National Centre for Text Mining (NaCTeM) and the Korea Institute of Science and Technology Information (KISTI). The entity annotation followed the automatically supported approach described above, and event annotation was performed fully manually by three biologists. The effort was organized by a coordinator with experience from several event annotation projects, but the individual annotators had no previous experience with the annotation of natural language texts. After a brief introduction to event annotation and training with the annotation tool and guidelines, the primary annotation was performed otherwise independently, but with a random 20% of documents provided to all three annotators to evaluate consistency, identify points of disagreement, and measure inter-annotator agreement. Following the completion of the primary manual annotation, consistency checking using automatic methods to detect potentially inconsistent annotations was performed similarly as in the CG task data preparation (described above).

An evaluation of the redundantly annotated documents after consistency checking indicated an inter-annotator agreement of 61.0% F-score for the event annotation using the primary task evaluation criteria. This level of agreement is somewhat low given the comparative simplicity of the task annotation targets. This can likely be attributed in substantial part to the facts that the annotators were working in a different country from the annotation coordinators, communicating primarily over email, and that only a relatively short period of annotator training could be included due to the overall project schedule. This serves to emphasize that while it is necessary to involve domain experts in annotation efforts targeting specialized texts, such expertise must be accompanied by thorough annotator training that provides sufficient experience on the use of the annotation formalism and tools as well as detailed understanding of the annotation guidelines. Finally, the redundantly annotated documents were assessed by the annotation coordinator to select the best of each set of annotations for the final corpus. This selection implies that the measured inter-annotator agreement of 61% F-score is a lower bound on the quality of the released PC task event annotation.

#### Corpus statistics

Both the CG and PC task corpora were divided into training, development and test sets representing approximately 50%, 17%, and 33% of the documents, respectively. Table 8 summarizes the CG corpus statistics, and Table 9 gives the statistics of the PC corpus. We note that the CG corpus is the largest of the BioNLP ST 2013 corpora by most of these measures, including in particular the number of annotated events. While the PC task corpus is somewhat smaller, it nevertheless has more annotated events than all corpora in previous BioNLP ST tasks with the exception of the original GE task. We thus expect that the availability of sufficient numbers of training examples should not be a limiting factor for the performance of systems participating in either task compared to previous BioNLP ST tasks.

**Table 8:** Cancer Genetics task corpus statistics.

Item	Train	Devel	Test	Total
Documents	300	100	200	600
Words	66082	21732	42064	129878
Entities	11034	3665	6984	21683
Relations	466	176	275	917
Events	8803	2915	5530	17248
Modifications	670	214	442	1326

**Table 9:** Pathway Curation task corpus statistics.

Item	Train	Devel	Test	Total
Documents	260	90	175	525
Words	53811	18579	35966	108356
Entities	7855	2734	5312	15901
Relations	455	128	330	913
Events	5992	2129	4004	12125
Modifications	317	80	174	571

### Evaluation

The evaluation of both the CG and PC tasks is instance-based and uses the standard precision, recall and F-score metrics. The primary criteria for determining if an event (or event modification) predicted by a participating system matches a gold standard event (modification) follow those first introduced in the initial BioNLP ST. In brief, these criteria require that a predicted and gold event are identical with two possible exceptions: the boundaries of predicted events may differ from those of the corresponding gold event by up to one word on each side, and the events that are referred to as arguments of the predicted and gold events under consideration may differ in their non-core arguments (see Tables 3 and 5). We refer to Kim et al. [28] for a detailed definition of these criteria. The statistical significance of the performance differences between each pair of participating systems was assessed using the approximate randomization method [62,63] using 9,999 repetitions.

We consider here also two variants of the primary evaluation setting: evaluation on *core *extraction targets only, and evaluation using an additional relaxation of the matching criteria termed *single partial penalty*. For the core task evaluation, we removed from both the gold annotation and the predictions of each participant all but the core event arguments (Tables 3 and 5) as well as event modifications, and then eliminated duplicate events arising from this simplification. This evaluation setting corresponds to the GE task subtask 1 [35] and the *core *evaluation settings of the EPI and Infectious Diseases (ID) tasks [36], focusing evaluation on a reduced set of targets defining only the minimum required to characterize an event. The single partial penalty criterion, first proposed in [64], aims to address the following issue in the primary evaluation criteria: events predicted by a system that otherwise match a gold standard event but either lack some of the arguments of that event or have some extra arguments are penalized twice: the predicted event counts as a false positive, and the gold standard event it largely corresponds to counts as a false negative. With the single partial penalty criterion, when a prediction and a gold standard event match partially in this way, either the prediction is counted as a false positive (extra arguments) or the gold standard event as a false negative (missing arguments), but not both. Finally, we consider also the combination of the core evaluation and single partial penalty variants.

## Results and discussion

### Participation

Final results for the two tasks were submitted by six teams, with all six submitting to the CG task and two submitting also to the PC task. Table 10 presents a summary of the teams, their members, and the ranks of their systems at the tasks they participated in. These teams involved members from six academic groups as well as one company, representing a broad variety of backgrounds, including linguists, bioinformaticians, and computer scientists among others.

**Table 10:** Participating teams, ranks and references to system descriptions.

Team	Institution	Tasks (rank)	Members	Ref
TEES-2.1	University of Turku	CG(1), PC(2)	1 BI	[94,95]
NaCTeM	National Centre for Text Mining	PC(1), CG(2)	1 NLP	[96,97]
NCBI	National Center for Biotechnology Information	CG(3)	3 BI	[98,99]
RelAgent	RelAgent Private Ltd.	CG(4)	1 LI, 1 CS	[71]
UET-NII	University of Engineering and Technology, Vietnam and National Institute of Informatics, Japan	CG(5)	6 CS	[100]
ISI	Indian Statistical Institute	CG(6)	2 ML, 2 NLP	-

Abbreviations: BI: Bioinformatician, CS: Computer Scientist, LI: Linguist, ML: Machine Learning researcher, NLP: Natural Language Processing researcher.

Table 11 summarizes the key properties of the systems applied to the two tasks. The participants approached the tasks using a broad variety of different architectures, including one-best machine learning pipelines (TEES-2.1 and NaCTeM), a joint subgraph matching-based approach (NCBI), a rule-based method (RelAgent), and two parsing-based approaches (UET-NII and ISI). Machine learning-based processing stages are dominated by the application of support vector machines, a popular learning method also in previous shared tasks.

**Table 11:** Summary of system architectures.

	NLP methods		Events				Resources	
**Team**	**Lexical**	**Syntactic**	**Trigger**	**Arg**	**Group**	**Modif.**	**Corpora**	**Other**

TEES-2.1	Porter	McCCJ + SD	SVM	SVM	SVM	SVM	GE	hedge words
NaCTeM	Snowball	Enju, GDep	SVM	SVM	SVM	SVM	(see text)	triggers
NCBI	MedPost, BLem	McCCJ + SD	Joint, subgraph matching			-	GE, EPI	-
RelAgent	Brill	fnTBL, custom	rules	rules	rules	rules	-	-
UET-NII	Porter	Enju	SVM	MaxEnt	Earley	-	-	triggers
ISI	CoreNLP	CoreNLP	NERsuite	Joint, MaltParser		-	-	-

Abbreviations: Trigger: event trigger detection, Arg: trigger-argument relation detection, Group: argument grouping into event structures, Modif.: event modification prediction, CoreNLP: Stanford CoreNLP, Porter: Porter stemmer, BLem: BioLemmatizer, Snowball: Snowball stemmer, McCCJ: McClosky-Charniak-Johnson parser, Charniak: Charniak parser, SD: Stanford Dependency conversion, SVM: Support Vector Machines, MaxEnt: Maximum Entropy Modeling.

A range of different methods was applied also in support of the individual processing stages. The methods used for basic word-level processing are generally comparatively simple, domain-independent approaches, with only the NCBI system using a lemmatization method developed specifically for domain texts (BioLemmatizer) [65]. By contrast, we note that all six systems perform syntactic analysis using primarily advanced, domain-specific parsing methods. These choices may be motivated in part by the observation that the application of parsers has been a very common feature of high-performing systems in previous BioNLP ST events. However, there is substantial variety in the specific choice of parser. The TEES-2.1 and NCBI systems use the McClosky-Charniak-Johnson constituency parser [66,67] and the Stanford Dependency conversion [68], which was the most common choice for syntactic analysis in BioNLP ST'11. The systems of NaCTeM and UET-NII use the probabilistic HPSG parser Enju [69], with NaCTeM additionally using the GDep shift-reduce dependency parser [70]. Finally, the Stanford CoreNLP tools are used by the ISI system, and a custom parser is applied by RelAgent [71].

Although event modifications were included as an extraction target in both tasks, three of the six systems involve no event modification component at all. Of the three that do, two apply a machine learning approach and one a rule-based approach. The choice of the three participants to exclude event modification from their methods may reflect at least in part the rarity of modifications compared to event annotations (see Tables 8 and 9) and the known difficulty of this subtask.

As in previous instantiations of the BioNLP ST, the 2013 shared task was run as an open challenge, where participants were encouraged to use any additional resources to augment the training data provided by the task organizers. Of such external resources, perhaps the most obviously applicable is the set of corpora introduced in previous shared tasks: as discussed in the introduction, these tasks have involved many of the same extraction targets, in particular basic biomolecular events. Three teams made use of one or more previously introduced BioNLP ST corpora. The TEES-2.1 and NCBI teams both used the GE corpus [35] and NCBI used also the EPI task corpus [36]. The NaCTeM system did not use any external corpora for the CG task, but for the PC task the system was applied with a stacked model [72] with predictions also from models trained on the BioNLP ST'11 GE, EPI and ID tasks [36] as well as from four event corpora not included in a shared task [24,73-75]. Three teams also applied lexical resources based on event corpora (Table 11).

We note that of the eight submissions received from the six participating groups, five represent applications or extensions of previously proposed approaches: the University of Turku and the National Centre for Text Mining submitted to both tasks using their established, state-of-the-art event extraction systems, the Turku Event Extraction System [76,77] (TEES) and EventMine [78,79] (NaCTeM), and the National Center for Biotechnology Information participated in the CG task using an extension of their previously proposed subgraph matching approach [80,81]. By contrast, the CG task submissions from the RelAgent, UET-NII and ISI teams used approaches that were now evaluated in a BioNLP ST event for the first time.

For more information on the participating systems, we refer to the descriptions of these systems published by the groups who created them, summarized in Table 10.

## Cancer Genetics primary evaluation results

The Cancer Genetics task primary evaluation results are presented in summary in Table 12. Statistical significance testing indicated that all pairwise differences between systems are significant (*p <*0.05) excepting for that between the NCBI and RelAgent systems.

**Table 12:** Cancer Genetics task primary evaluation result summary.

Team	recall	**prec**.	F-score
TEES-2.1	48.76	**64.17**	**55.41**
NaCTeM	**48.83**	55.82	52.09
NCBI	38.28	58.84	46.38
RelAgent	41.73	49.58	45.32
UET-NII	19.66	62.73	29.94
ISI	16.44	47.83	24.47

The TEES-2.1 system achieved the best performance at the task, averaging an F-score of 55%. A version of the same system ranked first also in the original 2009 BioNLP ST, then achieving an F-score of 52% [82], as well as in four out of eight tasks in the 2011 shared task, there achieving an F-score of 53% in the comparable GE task [83]. The NaCTeM group ranked second at the CG task, applying the EventMine system that has also been evaluated on numerous previous shared task corpora. The performance it achieves here, an F-score of 52%, is likewise at a broadly similar level to its performance at previously introduced tasks [84]. Also the third-ranking group, NCBI, extends on a system previously applied at the BioNLP ST'11, here performing notably better in relative terms at an F-score of 46% than the F-score of 41% achieved in the 2011 GE task [80].

Of the three newly introduced systems, the rule-based system of RelAgent achieves the best performance, averaging an F-score of 46% and thus performing within 10 percentage points of the top-ranking system. This shows that the CG task targets are not so complicated as to make the task inaccessible to rule-based approaches and that newly introduced systems can achieve a level of performance that is broadly competitive with the established state-of-the-art systems. The parsing-based systems achieve less competitive results, with F-scores of 30% for UET-NII and 24% for ISI. The results for both systems are primarily limited by low levels of recall, suggesting that better F-scores could perhaps be achieved by trading off some of the comparatively high precision.

We thus observe that the top three positions are taken by previously established systems - perhaps not a surprising finding - and, more importantly, that each of these systems achieves a level of performance at the CG task that is broadly comparable or better than the performance of the same system at previous related and narrower tasks such as GE. This is highly encouraging as it indicates that the state-of-the-art systems generalize to meet all of the novel challenges of the CG task, including the substantially increased number of entity and event types, the cancer domain, as well as the inclusion of higher levels of biological organization.

Table 13 gives the primary results separately for each event type. These results show that the newly introduced *anatomical *and *pathological *event categories are not particularly challenging for the event extraction methods; indeed, the best results for anatomical category events are better than the best results for *molecular *events (F-score of 77% versus 73%). The best results for pathological processes are only slightly lower at an F-score of 68%. By contrast to the broad new categories of events, the specific newly introduced event type Planned process PLANNED PROCESS proved challenging to extract (best result was an F-score of 41%), perhaps in part due to the fact that it frequently involves multiple arguments.

**Table 13:** Cancer Genetics task primary evaluation F-scores by event type.

Event	TEES-2.1	NaCTeM	NCBI	RelAgent	UET-NII	ISI
DEVELOPMENT	**71.43**	64.77	67.33	66.31	61.72	53.66
BLOOD VESSEL DEVELOPM	**85.28**	78.82	81.92	79.60	21.49	13.56
GROWTH	75.97	59.85	66.67	**76.92**	70.87	65.52
DEATH	**81.74**	73.17	74.07	64.71	77.78	63.16
CELL DEATH	73.30	75.18	**78.05**	66.98	25.17	7.35
CELL PROLIFERATION	**80.00**	78.33	72.73	64.39	71.43	57.40
CELL DIVISION	0.00	0.00	0.00	0.00	0.00	0.00
CELL DIFFERENTIATION	56.34	48.48	48.98	54.55	**59.26**	24.14
REMODELING	30.00	22.22	21.05	**40.00**	20.00	23.53
REPRODUCTION	100.00	100.00	100.00	100.00	100.00	100.00
*Anatomical total*	**77.20**	71.31	73.68	70.82	50.04	38.86
MUTATION	38.00	**41.05**	25.11	27.36	27.91	9.52
CARCINOGENESIS	**77.94**	72.18	67.14	64.12	35.96	24.72
CELL TRANSFORMATION	81.56	**82.54**	71.13	67.07	57.14	32.39
BREAKDOWN	**76.74**	70.13	76.54	42.42	58.67	50.70
METASTASIS	**70.91**	51.05	52.69	47.79	56.41	26.20
INFECTION	69.57	**76.92**	69.23	33.33	11.76	0.00
*Pathological total*	**67.51**	59.78	54.19	48.14	46.90	25.17
METABOLISM	**83.87**	70.27	74.29	80.00	68.75	71.43
SYNTHESIS	**78.26**	71.11	**78.26**	53.57	64.71	48.65
CATABOLISM	**63.64**	36.36	38.10	23.08	20.00	36.36
GLYCOLYSIS	0.00	**100.00**	95.45	97.78	0.00	0.00
AMINO ACID CATABOLISM	0.00	**66.67**	**66.67**	**66.67**	0.00	0.00
GENE EXPRESSION	78.21	**79.96**	73.69	69.45	58.01	53.28
TRANSCRIPTION	37.33	42.86	**51.55**	28.12	32.00	20.93
TRANSLATION	**40.00**	22.22	0.00	0.00	0.00	0.00
PROTEIN PROCESSING	**100.00**	**100.00**	**100.00**	0.00	**100.00**	**100.00**
ACETYLATION	**100.00**	**100.00**	66.67	**100.00**	66.67	66.67
GLYCOSYLATION	100.00	100.00	100.00	100.00	100.00	100.00
PHOSPHORYLATION	63.33	**70.37**	53.12	64.15	58.33	50.00
UBIQUITINATION	**100.00**	**100.00**	0.00	**100.00**	0.00	100.00
DEPHOSPHORYLATION	0.00	80.00	**100.00**	**100.00**	0.00	0.00
DNA METHYLATION	**66.67**	**66.67**	30.30	42.11	32.43	33.33
DNA DEMETHYLATION	0.00	0.00	0.00	0.00	0.00	0.00
PATHWAY	**71.30**	59.07	51.14	34.29	18.31	35.64
*Molecular total*	**72.60**	72.77	67.33	60.72	49.35	46.70
BINDING	**45.35**	43.93	37.89	32.69	33.94	11.92
DISSOCIATION	0.00	0.00	0.00	0.00	0.00	0.00
LOCALIZATION	54.83	**57.20**	47.58	45.22	44.94	35.94
*General total*	52.20	**53.08**	44.70	40.89	41.76	29.59
REGULATION	**32.66**	28.73	14.19	26.48	5.51	4.57
POSITIVE REGULATION	**45.89**	44.18	34.70	38.40	13.00	12.33
NEGATIVE REGULATION	**47.79**	43.17	33.20	40.47	10.30	12.16
*Regulation total*	**43.08**	39.79	29.21	35.58	10.30	10.29
PLANNED PROCESS	39.43	**40.51**	34.28	28.57	22.74	21.22
*Sub-total*	**56.75**	53.50	48.56	46.37	31.72	25.90

NEGATION	**40.00**	29.55	0.00	34.64	0.00	0.00
SPECULATION	27.14	**30.35**	0.00	25.90	0.00	0.00
*Modification total*	**34.66**	29.95	0.00	30.88	0.00	0.00

*Total*	**55.41**	52.09	46.38	45.32	29.94	24.47

The previously established event categories *general *and *regulation *remain as challenging here as in previous related challenges, likely reflecting at least partly the known challenges in the extraction of events with multiple arguments (e.g. BINDING) and those that recursively include other events (regulation types). Results are comparatively low also for the modification types, with a best F-score of 40% for NEGATION and 30% for SPECULATION. The extraction of these types involve challenges similar to those for regulation events in that their correct extraction requires also the correct extraction of the events that they apply to [28].

## Pathway Curation primary evaluation results

The overall results of the PC task are given in Table 14. The NaCTeM team achieves the better performance on the task, an F-score of 53%, using the EventMine system. The TEES-2.1 system comes in a close second at an F-score of 51%. Although these F-scores do not differ much, the two systems arrive at these results with quite different predictions: while the NaCTeM system exhibits balanced precision and recall, the performance of TEES-2.1 is noticeably skewed in favor of higher precision and lower recall.

**Table 14:** Pathway Curation task primary evaluation result summary.

Team	recall	**prec**.	F-score
NaCTeM	52.23	53.48	52.84
TEES-2.1	47.15	55.78	51.10

Table 15 shows the results of the PC task separated by event type. The two systems show very similar patterns of performance in terms of F-scores, again with the NaCTeM approach reaching its results with reasonably balanced precision and recall results for most types and TEES-2.1 frequently showing a clear preference for precision over recall. The performance for different event categories shows mainly similar patterns as in previous molecular-level event extraction tasks: *simple *modification events such as PHOSPHORYLATION that frequently take only a single *Theme *argument are comparatively easy extraction targets (a better result of an F-score of 66%), while structures that require the correct extraction of events that they apply to are challenging (40% F-score for *regulation *and 29% for *modifications*). Interestingly, the *general *category of events, and in particular the previously often particularly demanding BINDING type show comparatively high results here, perhaps reflecting some special characteristics of the domain.

**Table 15:** Pathway Curation task primary evaluation results by event type.

Event	NaCTeM			TEES-2.1		
	**recall**	**prec**.	**F-score**	**recall**	**prec**.	**F-score**

CONVERSION	34.33	35.48	34.90	35.82	42.86	**39.02**
PHOSPHORYLATION	62.46	55.94	59.02	53.40	66.00	**59.03**
DEPHOSPHORYLATION	45.00	56.25	**50.00**	35.00	77.78	48.28
ACETYLATION	69.57	72.73	71.11	82.61	76.00	**79.17**
DEACETYLATION	33.33	33.33	**33.33**	0.00	0.00	0.00
METHYLATION	42.86	60.00	50.00	57.14	80.00	**66.67**
DEMETHYLATION	100.00	100.00	100.00	100.00	100.00	100.00
UBIQUITINATION	52.94	64.29	58.06	58.82	76.92	**66.67**
DEUBIQUITINATION	100.00	100.00	100.00	100.00	100.00	100.00
LOCALIZATION	42.25	61.22	**50.00**	43.66	54.39	48.44
TRANSPORT	65.52	61.29	**63.33**	56.55	59.85	58.16
GENE EXPRESSION	90.65	83.15	**86.74**	84.55	79.39	81.89
TRANSCRIPTION	71.15	82.22	**76.29**	57.69	73.17	64.52
TRANSLATION	0.00	0.00	0.00	50.00	100.00	**66.67**
*Simple-total*	66.42	64.80	**65.60**	60.40	67.87	63.92
DEGRADATION	78.57	89.19	**83.54**	78.57	78.57	78.57
ACTIVATION	78.54	70.96	**74.56**	72.06	72.06	72.06
INACTIVATION	44.62	55.77	**49.57**	38.46	45.45	41.67
BINDING	64.96	47.30	**54.74**	53.96	53.96	53.96
DISSOCIATION	38.46	46.88	**42.25**	35.90	45.16	40.00
PATHWAY	84.91	75.50	**79.93**	70.94	75.50	73.15
*General-total*	69.07	62.69	**65.72**	61.16	65.74	63.37
REGULATION	33.33	33.97	33.65	29.73	39.51	**33.93**
POSITIVE REGULATION	35.49	42.81	38.81	34.51	45.45	**39.23**
NEGATIVE REGULATION	45.75	50.64	**48.07**	41.02	47.37	43.97
*Regulation-total*	37.73	42.79	**40.10**	35.17	44.76	39.39
*Sub-total*	53.47	53.96	**53.72**	48.23	56.22	51.92

NEGATION	24.52	35.87	29.13	25.16	41.30	**31.27**
SPECULATION	15.79	22.22	**18.46**	0.00	0.00	0.00
*Modification-total*	23.56	34.65	28.05	22.41	40.00	**28.73**

*Total*	52.23	53.48	**52.84**	47.15	55.78	51.10

It is interesting that the ranks of the two systems are inverted here compared to the CG task, where the TEES-2.1 system achieved 3 percentage points higher performance than EventMine. The only notable difference in the way that the systems were applied in the two tasks is that the EventMine system was trained using a large number of previously introduced resources for the PC but not the CG task [72]. Although the differences in relative performance may be due in part also to other factors, this is a promising indication that the use of previously introduced event resources in training may be beneficial for this extraction task despite its differences from those resources.

As discussed in detail above, both EventMine and TEES are well-established, support vector machine-based pipeline systems that have demonstrated state-of-the-art performance across many previous event extraction tasks [78,83]. Thus, even though the PC task attracted only limited participation at the BioNLP ST'13, the finding that these two highly competitive systems perform in the 51-53% F-score range suggests that this is probably a reasonable estimate of the best performance that current event extraction systems can achieve at this task.

It is perhaps somewhat surprising that the best results for this purely molecular-level task are lower than those for the considerably broader CG task despite the same systems achieving the best results in both. However, it is nevertheless a positive finding that this newly introduced task employing a novel reference ontology and aligned with a representation that has not previously been directly applied in event extraction task can be addressed with established technology without clear decreases in performance compared to established tasks [35].

## Additional evaluation results

We next present evaluation results under different variants of the evaluation criteria. Note that the supplementary evaluation results presented in this section do not supersede the primary evaluation results (Tables 12 and 14), which remain the official results of the shared task.

Table 16 shows the CG task results for the core extraction targets. The F-score results show improvements ranging between 2.6-4.7% points for the various systems, reflecting the comparative simplicity of the core targets and the frequency of non-core targets: approximately 8% of test set events involve event modifications, i.e. NEGATION or SPECULATION annotations, and 6% involve non-core arguments. The greatest benefit is observed for the NCBI system, which unlike the highest-ranking two systems does not attempt to predict event modifications.

**Table 16:** Cancer Genetics task core evaluation results.

Team	recall	**prec**.	F-score	Δ*_f_*
TEES-2.1	52.14	66.18	58.33	2.92
NaCTeM	53.32	58.98	56.01	3.92
NCBI	43.33	62.07	51.04	4.66
RelAgent	44.82	52.40	48.32	3.00
UET-NII	22.08	65.21	33.00	3.06
ISI	18.57	49.93	27.08	2.61

The Δ*_f _*column shows absolute difference to the primary evaluation F-score.

Table 17 gives the core task results for the Pathway Curation task. The observed effect is more modest than for the CG task, likely reflecting both the lower frequency of non-core targets (4% of events have modifications, 6% non-core arguments) and the fact that both participating systems predict modifications and all event arguments.

**Table 17:** Pathway Curation task core evaluation results.

Team	recall	**prec**.	F-score	Δ*_f_*
NaCTeM	54.14	54.78	54.46	1.62
TEES-2.1	49.49	57.02	52.99	1.89

The Δ*_f _*column shows absolute difference to the primary evaluation F-score.

Table 18 summarizes the evaluation results for the CG task with the single partial penalty criterion. For the full task, this criterion provides for most systems comparable F-scores as evaluation on the core targets only (Table 16), although with a different precision/recall balance. Evaluation with the single partial penalty criterion on the core targets shows that the relative improvements in F-score from these two variants of the primary evaluation are nearly additive, indicating that the variants address different aspects of the task.

**Table 18:** Cancer Genetics task evaluation results with single partial penalty.

	Primary (full task)				Core			
**Team**	**recall**	**prec.**	**F-score**	**Δ*_f_***	**recall**	**prec.**	**F-score**	**Δ*_f_***

TEES-2.1	50.64	68.78	58.33	2.92	53.72	70.49	60.97	2.64
NaCTeM	50.70	61.88	55.74	3.65	55.03	64.43	59.36	3.35
NCBI	39.75	65.97	49.61	3.23	44.35	68.54	53.85	2.81
RelAgent	43.47	54.39	48.32	3.00	46.55	57.21	51.33	3.01
UET-NII	22.35	67.01	33.52	3.58	24.85	68.65	36.49	3.49
ISI	17.65	51.17	26.25	1.78	19.92	52.96	28.95	1.87

The Δ*_f _*columns show absolute difference to the corresponding F-scores without single partial penalty.

The Pathway Curation task results with single partial penalty are shown in Table 19. For this task, the effect of this relaxed evaluation criterion is somewhat greater than that of considering core targets only, yet more limited than for the CG task. However, as for the CG task, the effects of the two variants are largely additive, with their combination providing a 4-5% point increase over the primary F-score results. We note that the effect of the single partial penalty criterion for the CG and PC task results broadly parallels its effect on the results of the BioNLP ST'11 EPI and ID tasks for which it was originally proposed [36].

**Table 19:** Pathway Curation task evaluation results with single partial penalty.

	Primary (full task)				Core Δ*_f_*			
**Team**	**recall**	**prec**.	**F-score**	**Δ*_f_***	**recall**	**prec**.	**F-score**	**Δ*_f_***

NaCTeM	54.14	57.02	55.54	2.70	56.12	58.34	57.21	2.75
TEES-2.1	49.66	58.77	53.83	2.73	52.13	59.85	55.72	2.73

The Δ*_f _*columns show absolute difference to the corresponding F-scores without single partial penalty.

### Discussion

**Figure 6:**
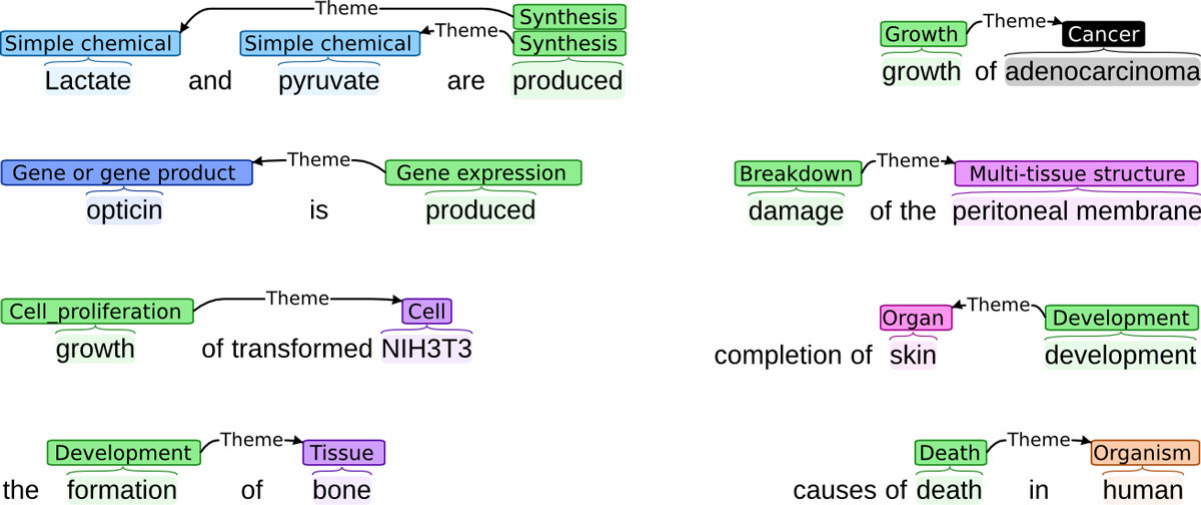
**Simple events**. Events with single arguments are reliably extracted regardless of factors such as text domain or level or biological organization.

We next summarize some of the main findings of the CG and PC task evaluations, consider specific challenges and possible ways of addressing these, and discuss the significance of the task results. Overall, we find that the best results in both the CG and PC tasks broadly parallel those achieved in similarly structured event extraction tasks in the BioNLP ST'09 [28] and '11 [35,36]. This suggests that the difficulty of the extraction task is not primarily determined by the factors that have varied between these tasks, such as the domain of the source texts (cancer, pathways, infectious diseases, etc.), the level of biological organization (molecular, cellular, tissue, etc.), the ontological basis applied in the annotation (GO, SBO), or, remarkably, even the number of different entity or event types targeted in the task. The detailed results bear out this observation (Tables 13 and 15). For example, we find that extraction performance for events taking only single arguments is comparatively high across the different categories outlined above (Figure 6).

**Figure 7:**
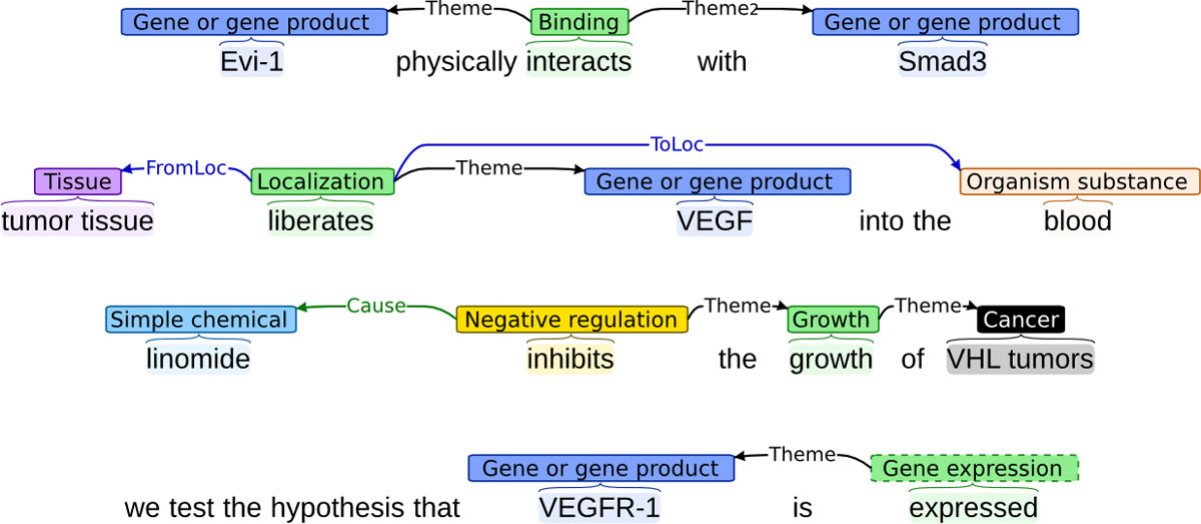
**Complex events**. Events involving multiple participants, recursive structure, and modifications continue to represent challenges for extraction.

Similarly, complex extraction targets such as events involving multiple arguments, events that recursively involve another event, and event modifications that often involve non-local "triggers" that are not explicitly annotated (Figure 7) represent challenges in all task settings. The relative difficulty of these extraction targets is well established, and much of it is inherent: it is more challenging to extract a structure involving three elements than one involving two, and the primary evaluation metrics of the shared tasks make no attempt to normalize away such effects. Nevertheless, the finding that most errors are found in these types of structures suggests that substantial advances in extraction performance may require extraction approaches emphasizing global features and joint models rather than the comparatively local approaches that currently dominate biomedical event extraction.

Previous detailed analyses of system errors [28,36] identified a number of specific properties that further characterize hard-to-extract events, including complex syntactic structure, participants identified through coreference, or reference to entities via relations such as *part-of*. Despite supporting tasks organized to address some of these challenges as part of the BioNLP ST'11 [85,86] and some successful implementations in event extraction systems [84], no system participating in the current tasks attempted integration of coreference resolution or entity relation extraction with event extraction. The combination of such analysis components is technically challenging, but it is likely that many events cannot be reliably extracted without such comprehensive integration.

It must also be noted that measured extraction performance is limited by the quality and consistency of the gold standard annotation: even domain experts trained to perform event annotation may reach no more than 60% F-score in cases, and 80% F-score remains a difficult target even for highly trained human annotators. The evaluation criteria can also be questioned: results using different criteria showed relative reductions in F-score error of 10% or more for most systems (Tables 18 and 19). These differences raise a question of which result is "correct", or which reflects the "true" performance of the systems. Naturally, no single number can adequately represent system performance in full, and preferences between different evaluation criteria are task-dependent and somewhat subjective. However, we note that the results of previous manual evaluations of system outputs (e.g. [36,87]) suggest that users may perceive the quality of system outputs as substantially higher than the primary evaluation results indicate, lending support to the application of also more relaxed criteria. Finally, we note that although evaluation variants produce substantial effects on absolute F-scores, all criteria agree on which of any two systems performs better. Thus, the primary results are stable in their ranking of systems by performance in both the CG and PC tasks.

## Conclusions

We have presented the Cancer Genetics and Pathway Curation tasks, two event extraction tasks that were newly introduced in the BioNLP Shared Task 2013. Both tasks are motivated by the needs of maintaining comprehensive and up-to-date information in the face of the enormous size and rapid growth of the biomedical domain scientific literature. For the CG task, such information can be used to develop knowledge bases on the molecular mechanisms underlying cancer; for the PC task, for developing, evaluating and maintaining molecular pathway models using representations such as SBML and BioPAX.

The two tasks both involve a number of aspects not previously considered in BioNLP ST settings. For the CG task, the most notable novel points are that the task addresses entities and events at all levels of biological organization from the molecular to the whole organism and involves pathological as well as physiological processes. The PC task stands out in particular in defining the structure of its extraction targets explicitly with reference to major pathway model representations and their types on the basis of the Systems Biology Ontology, thus aligning the extraction task closely with the needs of pathway curation efforts. Each of the tasks introduces a new, manually annotated corpus substantially extending on previously available resources. Both corpora draw their texts from PubMed abstracts, with the CG corpus containing annotations of over 17,000 events in 600 documents and the PC corpus over 12,000 events in 525 documents.

Six groups participated in the CG task, applying a broad range of extraction approaches including machine learning-based pipelines, a joint pattern matching-based approach, a rule-based approach and two parsing-based approaches. Two groups participated in the PC task, both applying well-established pipeline systems using support vector machines. All participating systems applied detailed analysis of sentence structure, most commonly in the form of full dependency parses. The best result achieved in the CG task was an F-score of 55.4% by the TEES-2.1 system, and the best result in the PC task was an F-score of 52.8% by the EventMine system. Both of the top-ranking systems are machine learning-based pipeline systems that have achieved state-of-the-art results in many previously proposed event extraction tasks.

The performance of the top-ranking systems at the two tasks is broadly in the range of the best results achieved at similar previously proposed event extraction tasks, indicating that current event extraction methods generalize well to meet the novel challenges represented by the CG and PC tasks.

Following the convention established in the first BioNLP Shared Task, both the Cancer Genetics and Pathway Curation tasks will continue as open challenges available to all interested participants. The corpora, evaluation tools, and supporting resources are available under open licenses from the BioNLP Shared Task homepage http://2013.bionlp-st.org/.

## Competing interests

The authors declare that they have no competing interests.

## Authors' contributions

SP and TO conceived of the tasks. SP coordinated the CG task organization, drafted the manuscript, and implemented evaluation and support tools. TO coordinated the PC task organization and led the CG and PC annotation efforts. RR and AR contributed to preparation of tools for the PC task. HC, SJ and SC contributed to the design and manual annotation of the PC task. SA participated in the design and coordination of both tasks and JT participated in the coordination of the overall shared task.
